# The genome sequence of the Common Yellow Sally, *Isoperla grammatica* (Poda, 1761)

**DOI:** 10.12688/wellcomeopenres.19066.1

**Published:** 2023-03-01

**Authors:** Emma McSwan, Caleala Clifford, Natural History Museum Genome Acquisition Lab, Darwin Tree of Life Barcoding collective, Wellcome Sanger Institute Tree of Life programme, Wellcome Sanger Institute Scientific Operations: DNA Pipelines collective, Tree of Life Core Informatics collective, Craig R. Macadam, Benjamin W. Price, Darwin Tree of Life Consortium

**Affiliations:** 1 Environment Agency, Chichester, UK; 2 Natural Resources Wales, Cardiff, Wales, UK; 3 Buglife – The Invertebrate Conservation Trust, Stirling, UK; 4 Life Science Department, Natural History Museum, London, UK

**Keywords:** Isoperla grammatica, Common Yellow Sally, genome sequence, chromosomal, Plecoptera

## Abstract

We present a genome assembly from an individual male *Isoperla grammatica* (the Common Yellow Sally; Arthropoda; Insecta; Plecoptera; Perlodidae). The genome sequence is 874.6 megabases in span. Most of the assembly is scaffolded into 14 chromosomal pseudomolecules, including the assembled X _1_ and X _2_ chromosomes. The mitochondrial genome has also been assembled and is 16.2 kilobases in length.

## Species taxonomy

Eukaryota; Metazoa; Ecdysozoa; Arthropoda; Hexapoda; Insecta; Pterygota; Neoptera; Polyneoptera; Plecoptera; Perloidea; Perlodidae; Isoperlinae; *Isoperla*; *Isoperla grammatica* (Poda, 1761) (NCBI:txid552050).

## Background

**Figure 1.**
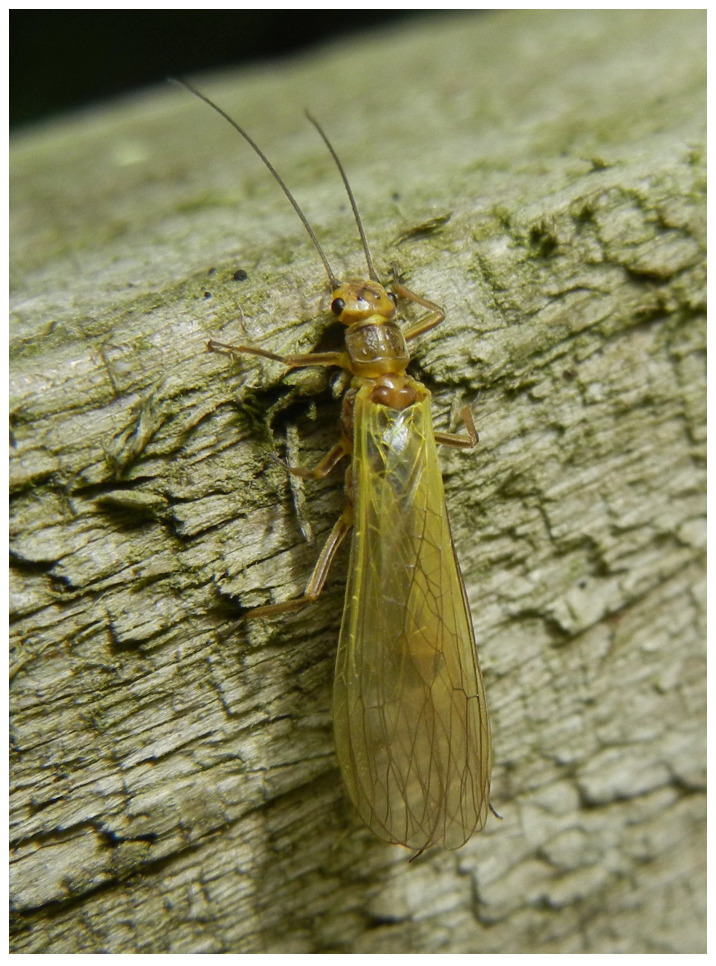
*Isoperla grammatica* © Jon Mortin (CC BY) Source: https://www.inaturalist.org/photos/146228992.

*Isoperla grammatica* ( Figure 1) is a western Palearctic species found across Europe from France to Romania, south to Sicily and north to the Baltic and Fennoscandia. It is found throughout Britain and Ireland and can be very common in some watercourses.

It is considered a eurytherm ( Graf *et al.*, 2009) and is typically occurs in high densities in all lotic water types with stable and unstable substrata, amongst moss, leaf packets and gravel, and is often present in rivers with slight organic enrichment ( Baars & Kelly-Quinn, 2006; Costello, 1988; Frost, 1942). The widespread distribution of this species indicates that it has no preference for particular pH conditions and has been found in both neutral and episodically acidic waters ( Feeley *et al.*, 2011; Feeley, 2012; Feeley & Kelly-Quinn, 2014; Murphy *et al.*, 2013).

*Isoperla grammatica* has a univoltine life cycle ( Frost, 1942; Smith *et al.*, 2000) with larvae present for part of two summers and the intervening winter ( Elliott, 1967; Langford, 1971; Malmqvist & Sjöström, 1989; Ulfstrand, 1968). Research in Norway and Britain indicated that eggs need warm temperatures of 7 to 12°C to initiate development, but the optimum incubation temperature is 16°C ( Elliott, 1991; Elliott, 1995; Lillehammer *et al.*, 1989; Saltveit & Lllehammer, 1984). Larvae occur all year round in small numbers and across various sizes, indicating variability in larval growth ( Langford, 1971; Malmqvist & Sjöström, 1989; Smith *et al.*, 2000). However, larvae typically grow rapidly in autumn and spring, although winter growth has also been noted where water temperatures were suitable ( Malmqvist & Sjöström, 1989).

Although diatom and algal matter are also ingested, the larvae of *I. grammatica* are carnivorous from very early instars ( Graf *et al.*, 2009; Malmqvist & Sjöström, 1989; Malmqvist *et al.*, 1991). Larvae are also highly selective in their prey items ( Williams, 1987). Of the prey items found, Chironomidae and Simuliidae seem to dominate ( Elliott, 2004; Malmqvist & Sjöström, 1989; Malmqvist *et al.*, 1991), with Williams highlighting a preference for Baetidae in Wales ( Williams, 1987). Elliott ( 2000, 2004) indicated that the feeding behaviour was by active search and was limited to the hours of dusk and dawn, with little activity during the day or at night. The adults feed on a range of pollens, fungi and fine particulate organic matter ( Tierno de Figueroa & Sánchez-Ortega, 1999).

The high-quality genome sequence described here is, to our knowledge, the first reported for *Isoperla grammatica*, and has been generated as part of the Darwin Tree of Life project. It will aid in understanding the biology, physiology and ecology of the species.

### Genome sequence report

The genome was sequenced from a male *Isoperla grammatica* specimen (ipIsoGram3) collected from River Test, Great Bridge, Hampshire (latitude 51.00, longitude –1.50). A total of 34-fold coverage in Pacific Biosciences single-molecule HiFi long reads was generated. Primary assembly contigs were scaffolded with chromosome conformation Hi-C data. Manual assembly curation corrected 400 missing or mis-joins and removed 47 haplotypic duplications, reducing the assembly length by 1.18% and the scaffold number by 24.31%, and increasing the scaffold N50 by 27.94%.

**Figure 2.**
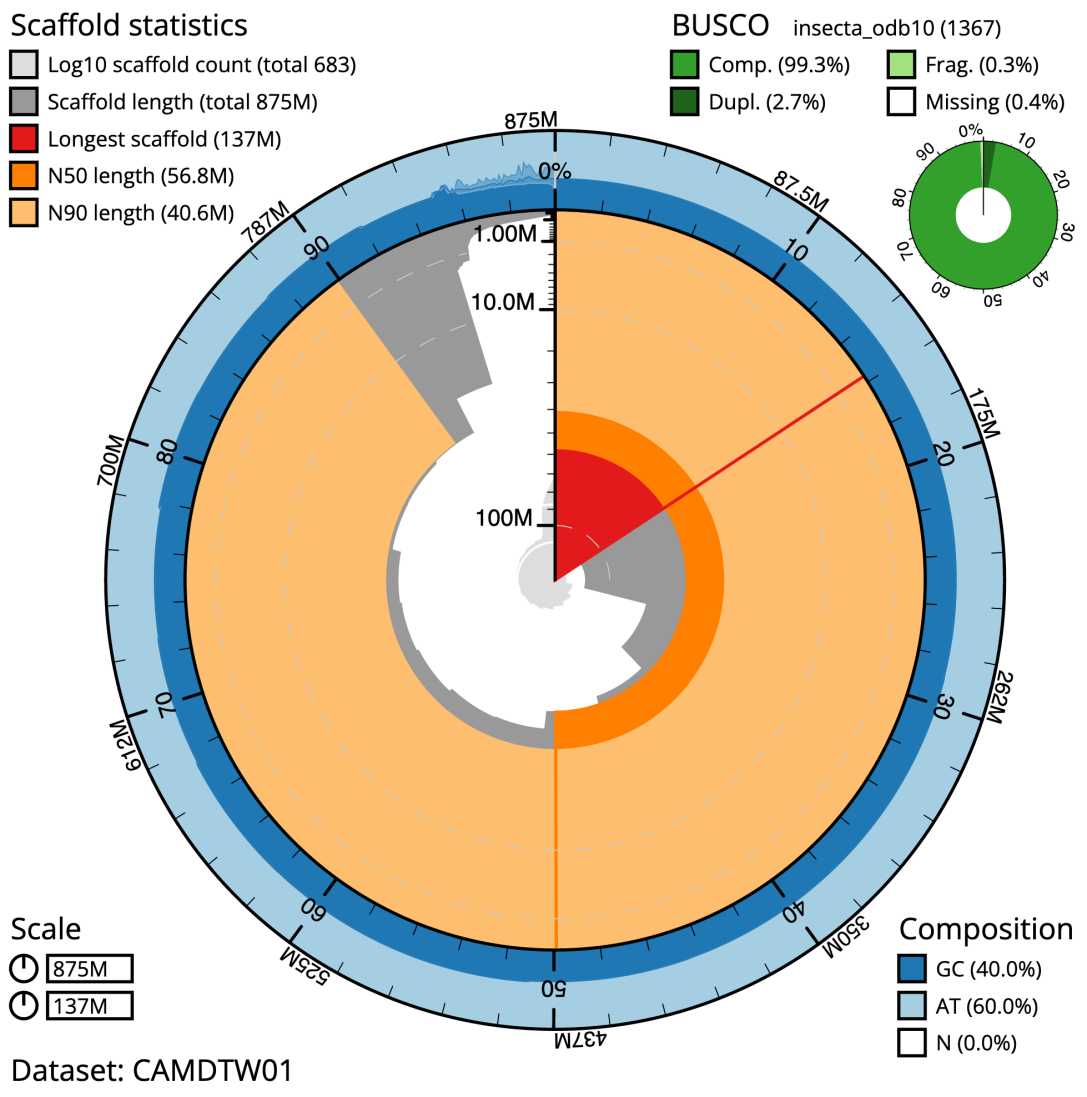
Genome assembly of *Isoperla grammatica*, ipIsoGram3.1: metrics. The BlobToolKit Snailplot shows N50 metrics and BUSCO gene completeness. The main plot is divided into 1,000 size-ordered bins around the circumference with each bin representing 0.1% of the 874,600,353 bp assembly. The distribution of scaffold lengths is shown in dark grey with the plot radius scaled to the longest scaffold present in the assembly (137,496,831 bp, shown in red). Orange and pale-orange arcs show the N50 and N90 scaffold lengths (56,757,646 and 40,559,246 bp), respectively. The pale grey spiral shows the cumulative scaffold count on a log scale with white scale lines showing successive orders of magnitude. The blue and pale-blue area around the outside of the plot shows the distribution of GC, AT and N percentages in the same bins as the inner plot. A summary of complete, fragmented, duplicated and missing BUSCO genes in the insecta_odb10 set is shown in the top right. An interactive version of this figure is available at https://blobtoolkit.genomehubs.org/view/ipIsoGram3.1/dataset/CAMDTW01/snail.

The final assembly has a total length of 874.6 Mb in 682 sequence scaffolds with a scaffold N50 of 56.8 Mb ( Table 1). Most (95.16%) of the assembly sequence was assigned to 14 chromosomal-level scaffolds, representing 12 autosomes, and the X _1_ and X _2_ sex chromosomes. Chromosome-scale scaffolds confirmed by the Hi-C data have been named in order of size. ( Figure 2– Figure 5; Table 2). The scaffold order and orientation are uncertain in the following regions: chromosome 8 (29.54–40.94 Mb), chromosome 9 (2.58–20.91 Mb), and chromosome 11 (24.65–31.18 Mb). The assembly has a BUSCO v5.3.2 ( Manni *et al.*, 2021) completeness of 99.3% (single 96.6%, duplicated 2.7%), using the insecta_odb10 reference set. While not fully phased, the assembly deposited is of one haplotype. Contigs corresponding to the second haplotype have also been deposited.

**Table 1.** Genome data for *Isoperla grammatica*, ipIsoGram3.1.

Project accession data		
Assembly identifier	ipIsoGram3.1	
Species	*Isoperla grammatica*	
Specimen	ipIsoGram3	
NCBI taxonomy ID	552050	
BioProject	PRJEB537291	
BioSample ID	SAMEA7521000	
Isolate information	ipIsoGram3 (PacBio) ipIsoGram4 (Hi-C) ipIsoGra76 (RNA-Seq)	
Assembly metrics *		*Benchmark*
Consensus quality (QV)	55.3	*≥ 50*
*k*-mer completeness	99.99%	*≥ 95%*
BUSCO **	C:99.3%[S:96.6%,D:2.7%], F:0.3%,M:0.4%,n:1,367	*C ≥ 95%*
Percentage of assembly mapped to chromosomes	95.16%	*≥ 95%*
Sex chromosomes	X1 and X2	*localised homologous pairs*
Organelles	Mitochondrial genome assembled	*complete single alleles*
Raw data accessions		
PacificBiosciences SEQUEL II	ERR9878387, ERR9878388	
Hi-C Illumina	ERR9881687	
PolyA RNA-Seq Illumina	ERR9881692	
Genome assembly		
Assembly accession	GCA_945910005.1	
*Accession of alternate haplotype*	GCA_945909985.1	
Span (Mb)	874.6	
Number of contigs	2,826	
Contig N50 length (Mb)	0.7	
Number of scaffolds	682	
Scaffold N50 length (Mb)	56.8	
Longest scaffold (Mb)	137.5	

* Assembly metric benchmarks are adapted from column VGP-2020 of “Table 1: Proposed standards and metrics for defining genome assembly quality” from ( Rhie *et al.*, 2021). ** BUSCO scores based on the insecta_odb10 BUSCO set using 5.3.2. C = complete [S = single copy, D = duplicated], F = fragmented, M = missing, n = number of orthologues in comparison. A full set of BUSCO scores is available at https://blobtoolkit.genomehubs.org/view/ipIsoGram3.1/dataset/CAMDTW01/busco.

**Figure 3.**
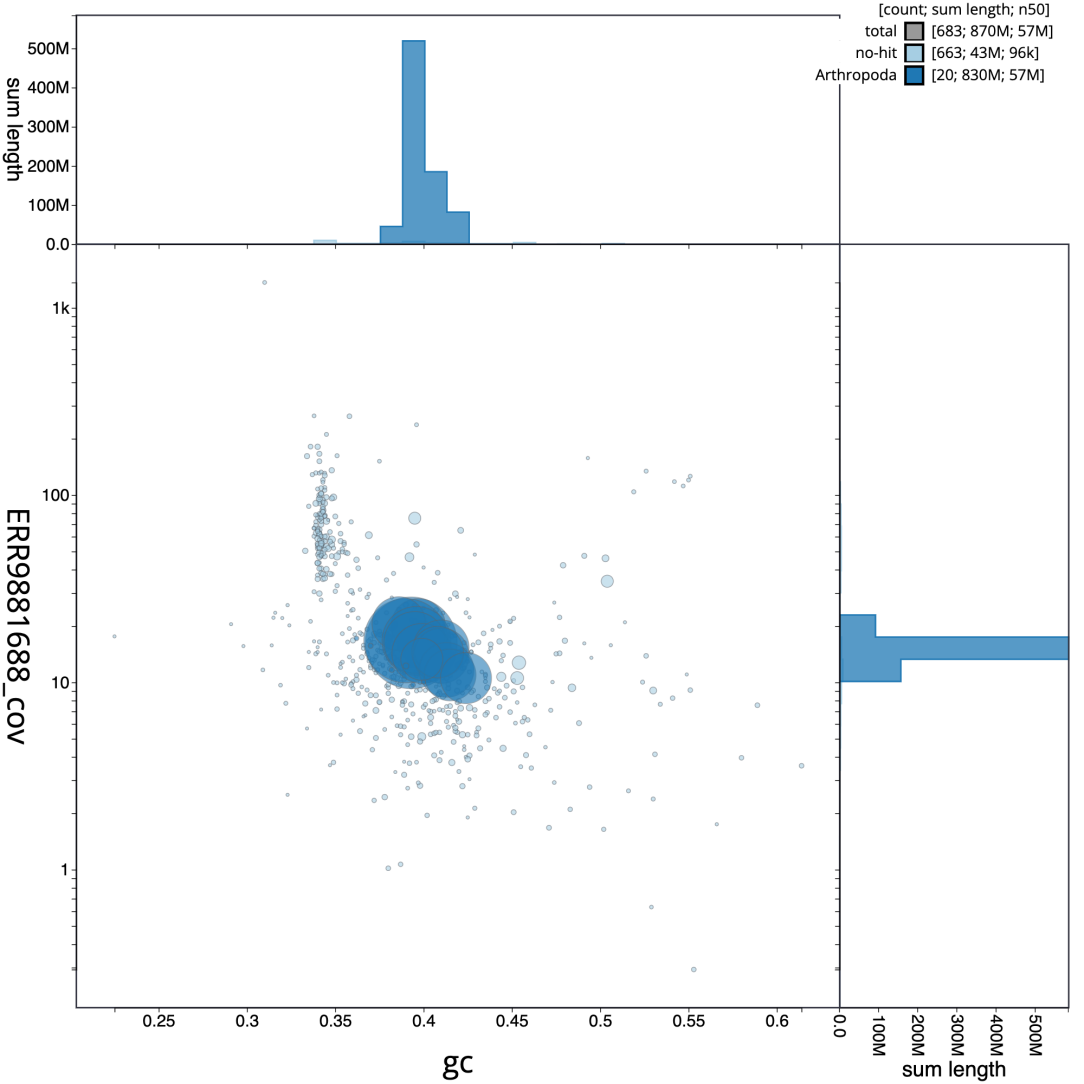
Genome assembly of *Isoperla grammatica*, ipIsoGram3.1: GC coverage. BlobToolKit GC-coverage plot. Scaffolds are coloured by phylum. Circles are sized in proportion to scaffold length. Histograms show the distribution of scaffold length sum along each axis. An interactive version of this figure is available at https://blobtoolkit.genomehubs.org/view/ipIsoGram3.1/dataset/CAMDTW01/blob.

**Figure 4.**
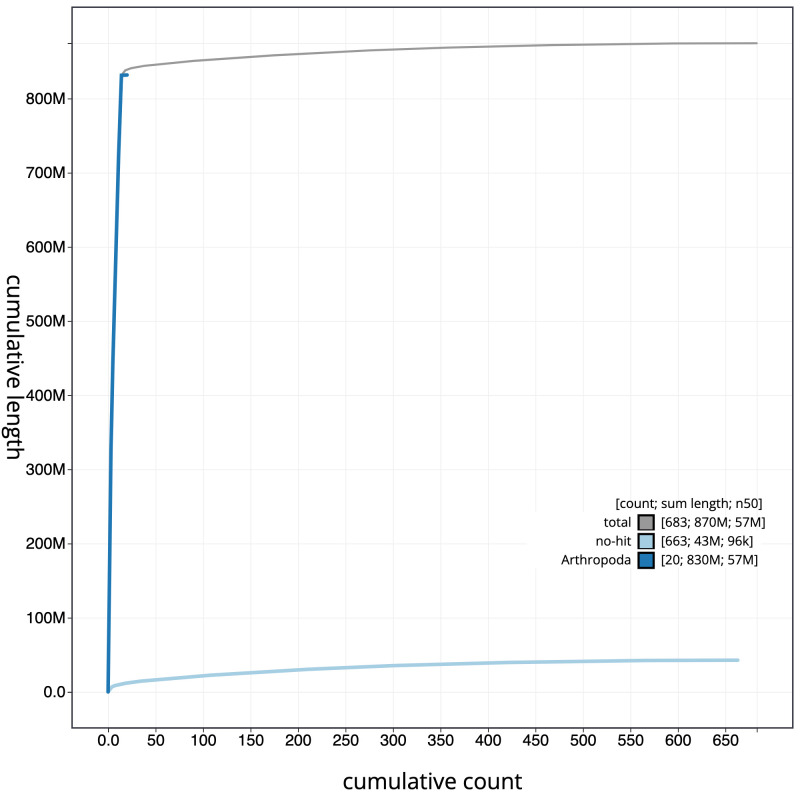
Genome assembly of *Isoperla grammatica*, ipIsoGram3.1: cumulative sequence. BlobToolKit cumulative sequence plot. The grey line shows cumulative length for all scaffolds. Coloured lines show cumulative lengths of scaffolds assigned to each phylum using the buscogenes taxrule. An interactive version of this figure is available at https://blobtoolkit.genomehubs.org/view/ipIsoGram3.1/dataset/CAMDTW01/cumulative.

**Figure 5.**
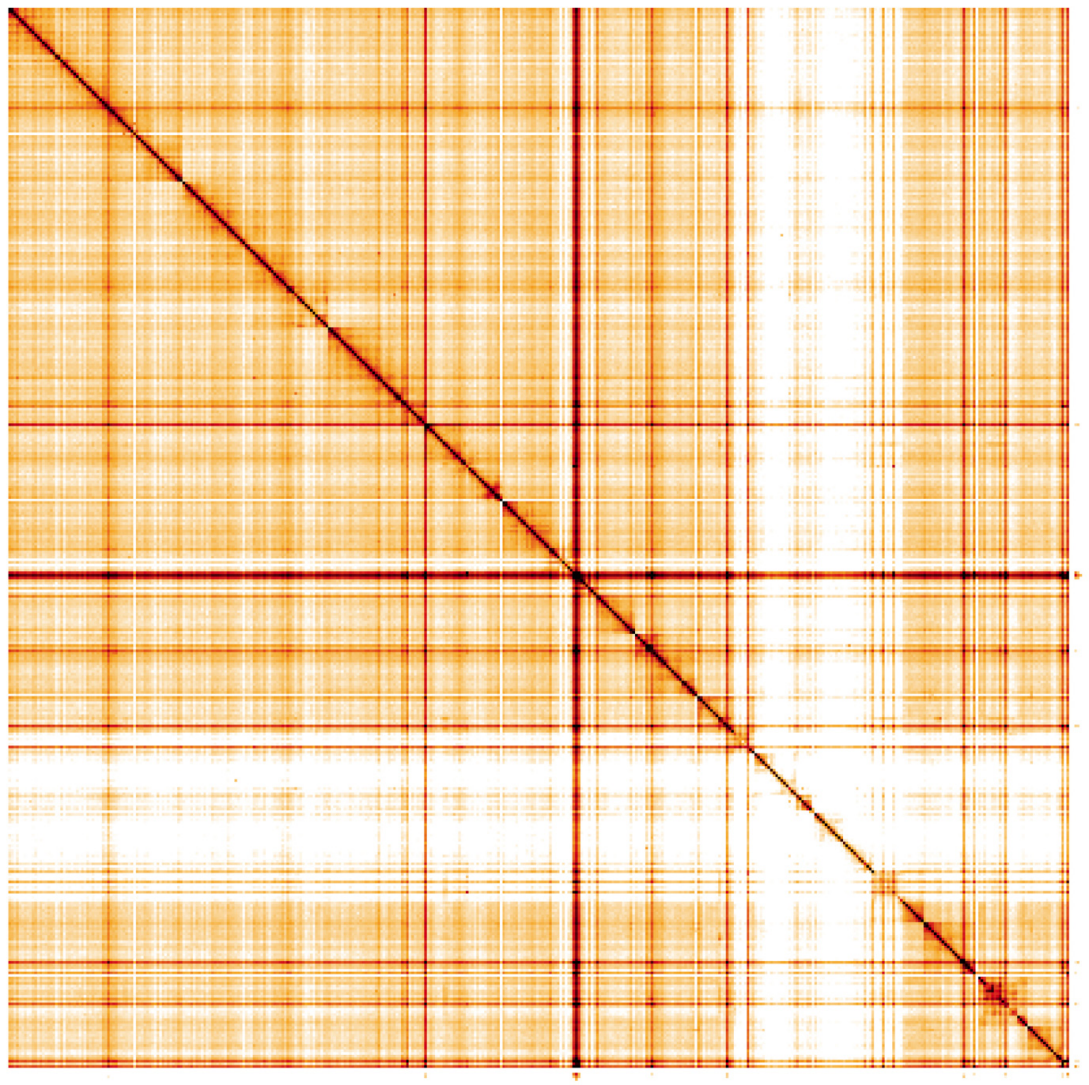
Genome assembly of *Isoperla grammatica*, ipIsoGram3.1: Hi-C contact map. Hi-C contact map of the ipIsoGram3.1 assembly, visualised using HiGlass. Chromosomes are shown in order of size from left to right and top to bottom. An interactive version of this figure may be viewed at https://genome-note-higlass.tol.sanger.ac.uk/l/?d=K-TmeZtbSuiS9poCrleNnA.

**Table 2.** Chromosomal pseudomolecules in the genome assembly of *Isoperla grammatica*, ipIsoGram3.

INSDC accession	Chromosome	Size (Mb)	GC%
OX246737.1	1	137.5	39.3
OX246745.1	X1	45.93	39.7
OX246738.1	2	115.38	39
OX246739.1	3	75.84	39.6
OX246746.1	X2	45.27	38.6
OX246740.1	4	60.58	39.5
OX246741.1	5	56.76	39.9
OX246742.1	6	48.65	41
OX246743.1	7	48.25	41
OX246744.1	8	46.81	41.3
OX246747.1	9	41.61	41.5
OX246748.1	10	41.44	40.8
OX246749.1	11	40.56	42.4
OX246750.1	12	26.74	39.9
OX246751.1	MT	0.02	31.3
-	unplaced	43.26	40

## Methods

### Sample acquisition and nucleic acid extraction

Two *Isoperla grammatica* specimens (ipIsoGram3 and ipIsoGram4) were collected from River Test, Great Bridge, Hampshire (latitude 51.00, longitude –1.50) on 19 March 2019. The specimens were taken from freshwater by Emma McSwan (Environment Agency) using a kick-net. The specimen was also identified by Emma McSwan and snap-frozen in a dry shipper at the Natural History Museum, London. A specimen used for RNA sequencing (ipIsoGram7) was collected by Caleala Clifford (Natural Resources Wales) from River Taff Fawr, Garwnant, UK (latitude 51.81, longitude –-3.44) on 19 March 2019 and snap-frozen in a dry shipper at the Natural History Museum, London.

DNA was extracted at the Tree of Life laboratory, Wellcome Sanger Institute (WSI). The ipIsoGram3 specimen was weighed and dissected on dry ice. The tissue was cryogenically disrupted to a fine powder using a Covaris cryoPREP Automated Dry Pulveriser, receiving multiple impacts. High molecular weight (HMW) DNA was extracted using the Qiagen MagAttract HMW DNA extraction kit. HMW DNA was sheared into an average fragment size of 12–20 kb in a Megaruptor 3 system with speed setting 30. Sheared DNA was purified by solid-phase reversible immobilisation using AMPure PB beads with a 1.8X ratio of beads to sample to remove the shorter fragments and concentrate the DNA sample. The concentration of the sheared and purified DNA was assessed using a Nanodrop spectrophotometer and Qubit Fluorometer and Qubit dsDNA High Sensitivity Assay kit. Fragment size distribution was evaluated by running the sample on the FemtoPulse system.

RNA was extracted from tissue of ipIsoGram7 in the Tree of Life Laboratory at the WSI using TRIzol, according to the manufacturer’s instructions. RNA was then eluted in 50 μl RNAse-free water and its concentration assessed using a Nanodrop spectrophotometer and Qubit Fluorometer using the Qubit RNA Broad-Range (BR) Assay kit. Analysis of the integrity of the RNA was done using Agilent RNA 6000 Pico Kit and Eukaryotic Total RNA assay.

### Sequencing

Pacific Biosciences HiFi circular consensus DNA sequencing libraries were constructed according to the manufacturers’ instructions. Poly(A) RNA-Seq libraries were constructed using the NEB Ultra II RNA Library Prep kit. DNA and RNA sequencing was performed by the Scientific Operations core at the WSI on Pacific Biosciences SEQUEL II (HiFi) and Illumina HiSeq 4000 (RNA-Seq). Hi-C data were also generated from ipIsoGram4 using the Arima v2 kit and sequenced on the NovaSeq 6000 instrument.

### Genome assembly

Assembly was carried out with Hifiasm ( Cheng *et al.*, 2021) and haplotypic duplication was identified and removed with purge_dups ( Guan *et al.*, 2020). The assembly was then scaffolded with Hi-C data ( Rao *et al.*, 2014) using YaHS ( Zhou *et al.*, 2023). The assembly was checked for contamination and corrected as described previously ( Howe *et al.*, 2021). Manual curation was performed using HiGlass ( Kerpedjiev *et al.*, 2018) and Pretext ( Harry, 2022). The mitochondrial genome was assembled using MitoHiFi ( Uliano-Silva *et al.*, 2022), which performed annotation using MitoFinder ( Allio *et al.*, 2020). The genome was analysed and BUSCO scores generated within the BlobToolKit environment ( Challis *et al.*, 2020). Table 3 contains a list of all software tool versions used, where appropriate.

**Table 3.** Software tools and versions used.

Software tool	Version	Source
BlobToolKit	3.4.0	Challis *et al.*, 2020
Hifiasm	0.16.1-r375	Cheng *et al.*, 2021
HiGlass	1.11.6	Kerpedjiev *et al.*, 2018
MitoHiFi	2	Uliano-Silva *et al.*, 2022
PretextView	0.2	Harry, 2022
purge_dups	1.2.3	Guan *et al.*, 2020
YaHS	yahs-1.1.91eebc2	Zhou *et al.*, 2023

### Ethics and compliance issues

The materials that have contributed to this genome note have been supplied by a Darwin Tree of Life Partner. The submission of materials by a Darwin Tree of Life Partner is subject to the Darwin Tree of Life Project Sampling Code of Practice. By agreeing with and signing up to the Sampling Code of Practice, the Darwin Tree of Life Partner agrees they will meet the legal and ethical requirements and standards set out within this document in respect of all samples acquired for, and supplied to, the Darwin Tree of Life Project. All efforts are undertaken to minimise the suffering of animals used for sequencing. Each transfer of samples is further undertaken according to a Research Collaboration Agreement or Material Transfer Agreement entered into by the Darwin Tree of Life Partner, Genome Research Limited (operating as the Wellcome Sanger Institute), and in some circumstances other Darwin Tree of Life collaborators.

## Data Availability

European Nucleotide Archive: *Isoperla grammatica* (common yellow sally). Accession number PRJEB53729; https://identifiers.org/ena.embl/PRJEB53729 ( Wellcome Sanger Institute, 2022) The genome sequence is released openly for reuse. The *Isoperla grammatica* genome sequencing initiative is part of the Darwin Tree of Life (DToL) project. All raw sequence data and the assembly have been deposited in INSDC databases. The genome will be annotated using available RNA-Seq data and presented through the Ensembl pipeline at the European Bioinformatics Institute. Raw data and assembly accession identifiers are reported in Table 1.

## Author information

Members of the Natural History Museum Genome Acquisition Lab are listed here: https://doi.org/10.5281/zenodo.4790043.

Members of the Darwin Tree of Life Barcoding collective are listed here: https://doi.org/10.5281/zenodo.4893703.

Members of the Wellcome Sanger Institute Tree of Life programme are listed here: https://doi.org/10.5281/zenodo.4783585.

Members of Wellcome Sanger Institute Scientific Operations: DNA Pipelines collective are listed here: https://doi.org/10.5281/zenodo.4790455.

Members of the Tree of Life Core Informatics collective are listed here: https://doi.org/10.5281/zenodo.5013541.

Members of the Darwin Tree of Life Consortium are listed here: https://doi.org/10.5281/zenodo.4783558.
