# Supplementary material for: The genome sequence of the Common Yellow Sally, Isoperla grammatica (Poda, 1761) (Plecoptera: Perlodidae)
Source: Wellcome Open Res. 2026 Mar 19;8:107. Originally published 2023 Mar 1. [Version 2] doi: 10.12688/wellcomeopenres.19066.2 (PMC13033136; doi:10.12688/wellcomeopenres.19066.2)
Supplement: Peer review history

# Reviewer report: Reviewer response for version 2

**DOI:** 10.21956/wellcomeopenres.23612.r151496

**Published:** 2026-04-08

**Authors:** Jinjun Cao

**Affiliations:** 1 Henan Institute of Science and Technology, Xinxiang, Henan, China

This data note presents a high-quality, chromosome-level genome assembly for *Isoperla grammatica* (Common Yellow Sally), a stonefly species found throughout Britain and Ireland. The paper includes detailed methods for sample collection, DNA/RNA extraction, sequencing, assembly, and quality assessment. Three specimens were collected from different locations in the UK in March 2019, with one used for the primary genome assembly.

 The description of certain key steps in the Methods section is overly general, which compromises the reproducibility of the study. The rigor of some result descriptions needs to be improved.

 1. For Mitochondrial genome assembly: The description of MitoHiFi in “Methods -> Genome assembly and curation” is too brief. Clarification should be added regarding the input data (e.g., whether they were extracted from preliminarily assembled contigs by Hifiasm or assembled de novo from raw HiFi reads), along with a brief explanation of the reference mitochondrial genome or parameter settings used for annotation.

 2. For Hi-C data mapping: The manuscript states that “Hi-C reads were mapped to the primary contigs using bwa-mem2” but provides no mapping parameters. Basic alignment parameters (such as default settings or specific parameters employed) should be supplemented, as this is standard practice.

Are sufficient details of methods and materials provided to allow replication by others?

Partly

Is the rationale for creating the dataset(s) clearly described?

Yes

Are the datasets clearly presented in a useable and accessible format?

Yes

Are the protocols appropriate and is the work technically sound?

Partly

Reviewer Expertise:

Phylogeny and Evolution of Plecoptera

I confirm that I have read this submission and believe that I have an appropriate level of expertise to confirm that it is of an acceptable scientific standard, however I have significant reservations, as outlined above.

# Reviewer report: Reviewer response for version 2

**DOI:** 10.21956/wellcomeopenres.23612.r151233

**Published:** 2026-03-28

**Authors:** R Edward DeWalt

**Affiliations:** 1 Illinois Natural History Survey, Champaign, Illinois, USA

This version I believe is ready to be accepted.

 A few improvements...

 1. A citation with my name should be DeWalt not Dewalt. It is correct in the reference.

 The sentence "It is considered a eurytherm (Graf et al., 2009) and is typically occurs..." should read "It is considered a eurytherm (Graf et al., 2009) and typically occurs...."

 Otherwise, I am happy with the revision.

Are sufficient details of methods and materials provided to allow replication by others?

No

Is the rationale for creating the dataset(s) clearly described?

No

Are the datasets clearly presented in a useable and accessible format?

Yes

Are the protocols appropriate and is the work technically sound?

Yes

Reviewer Expertise:

Plecoptera taxonomy and systematics.

I confirm that I have read this submission and believe that I have an appropriate level of expertise to confirm that it is of an acceptable scientific standard.

# Reviewer report: Reviewer response for version 1

**DOI:** 10.21956/wellcomeopenres.21139.r59815

**Published:** 2023-07-03

**Authors:** R Edward DeWalt

**Affiliations:** 1 Illinois Natural History Survey, Champaign, Illinois, USA

The authors present a genome sequence for the stonefly Isoperla grammatica. There is little to no rationale provided for this work. What is the Darwin Tree of Life project? How is the dataset important? How will it used?

 I assume that the identity of the specimens used is *I. grammatica*. Craig Macadam can certain identify this species, but he is not listed as the identifier nor is it said he confirmed the identification by McSwan. It would be good to state that this basic effort has been done to ensure the identity of the specimens use. I am disturbed by the total lack of voucher material. The presence of vouchers is a basic tenant of taxonomic science and without it repeatability is compromised! Not even photo vouchers are presented. The image of the adult provided is only an exemplar and matches no specimens sequenced in this work.

 While this may be the only instance of high quality genome sequences for *I. grammatic*, other work using transcriptome and whole genome sequencing have recently been published (South *et al*. 2021a ^1^ & b ^2^, Letch *et al.* 2021 ^3^). These should be cited. One of the other reviewers asked for a reference for the distribution of the this species. DeWalt *et al*. 2023 could be used.

 One other reviewer thought the biology section was too long. I don't agree.

Are sufficient details of methods and materials provided to allow replication by others?

No

Is the rationale for creating the dataset(s) clearly described?

No

Are the datasets clearly presented in a useable and accessible format?

Yes

Are the protocols appropriate and is the work technically sound?

Yes

Reviewer Expertise:

Plecoptera taxonomy and systematics.

I confirm that I have read this submission and believe that I have an appropriate level of expertise to confirm that it is of an acceptable scientific standard, however I have significant reservations, as outlined above.

## References

[1] : A New Family of Stoneflies (Insecta: Plecoptera), Kathroperlidae, fam. n., with a Phylogenomic Analysis of the Paraperlinae (Plecoptera: Chloroperlidae). *Insect Systematics and Diversity* .2021;5(4) : 10.1093/isd/ixab014 10.1093/isd/ixab014

[2] : Combining molecular datasets with strongly heterogeneous taxon coverage enlightens the peculiar biogeographic history of stoneflies (Insecta: Plecoptera). *Systematic Entomology* .2021;46(4) : 10.1111/syen.12505 952-967 10.1111/syen.12505

[3] : Phylogenomics of the North American Plecoptera. *Systematic Entomology* .2021;46(1) : 10.1111/syen.12462 287-305 10.1111/syen.12462

# Response

**Published:** 2025-09-24

**Authors:** Tree of Life Team Sanger

**Affiliations:** Wellcome Trust Sanger Institute, UK

Thank you for reviewing this data note. We have resubmitted a new version and have found your comments helpful. Responses to some of your points are below: 

 1. Specimen identification. 

 We are unable to supply a voucher photograph, although specimen identification was done using reliable keys. We did a BOLD barcode check on the DNA during assembly, and the results are available on the TOLQC page (also now linked in the article): 

 https://tolqc.cog.sanger.ac.uk/darwin/insects/Isoperla_grammatica/

 2. Other work using molecular data. 

 We have expanded on this in the introduction. 

 The Tree of Life team

# Reviewer report: Reviewer response for version 1

**DOI:** 10.21956/wellcomeopenres.21139.r59818

**Published:** 2023-06-22

**Authors:** Tilman Schell

**Affiliations:** 1 LOEWE Centre for Translational Biodiversity Genomics (LOEWE-TBG), Frankfurt am Main, Hessen, Germany

Comments to the authors:

 McSwan *et al.* present the de novo genome assembly of Isoperla grammatica. PacBio HiFi reads and Arima Hi-C data were utilized to achieve chromosome level scaffolds. The presented analyses show a high quality of the assembly.

 The manuscript is well written and methods are described in a way to ensure reproducibility. Nevertheless, I do have some remarks that need to be clarified before indexing.

Major remarks:

 Not sure if this applies more to the journal formatting but without line numbers pointing to certain locations in the text.

 In the section “Genome sequence report” you state that the scaffolds represent chromosomes. Is there any previous work showing the karyotype of this species? If so, you would need to state this here. Otherwise you should be more careful by stating that the chromosome-level scaffolds are probably representing mostly complete chromosomes based on Hi-C data.

 Additionally, using “(chromosome-scale) scaffolds” and “chromosomes” synonymous, is not applicable as long as you could e.g. proof correspondence of assembled sequence and actual molecules by staining experiments.

 Is there genome size estimate of this species? It further underlines the quality of an assembly, if total assembly length and estimated genome size are similar.

 It would be helpful to state the total HiFi Gbp and HiFi N50s of the different SMRT cells. I assume that there are multiple SMRT cells sequenced as you state “Pacific Biosciences HiFi circular consensus DNA sequencing libraries [...]” in the “Sequencing” section and there are two accession numbers in Table 1. Please clarify this a bit more in the main text.

 The methods on mt genome assembly are very brief, please describe this in more detail. Did you used contigs or reads as input? Did you filter out contigs representing (parts of the) mt genome?

 Mapping of Hi-C reads is not described at all. Please add this.

Minor remarks:

 General:

 Please label y-axis to be readable from the right.

 Just to be sure: You created RNAseq data but you did not use it in this study, or?

 What parameters were used in the applied tools? All were running with default settings? Please add this to the methods.

 Particular:

 Table 1: The QV value is presented here but it is not described how this value was computed. Please add this to the methods.

 “Percentage of assembly mapped to chromosomes”: Probably you mean “anchored in scaffolds”.

 How were the sex chromosomes (better: scaffolds representing sex chromosomes) identified? This is not described.

 Figure 3: “GC-coverage plot”: As far as I am aware this is called “blob plot”.

 Was there any filtering involved here? If yes, you should state this in the methods. If not it looks surprisingly clean, as you state a whole (?) specimen was used for DNA extraction.

 Did you check the blob plot before scaffolding? In the worst case there are parts of contaminants scaffolded into the larger scaffolds and finally not visible anymore.

 Figure 5: As the other reviewer already stated, it is not very constructive to show a contact map without showing the scaffold and contig boundaries.

 Are the two scaffolds representing the sex chromosomes in the area with the reduced signal? Maybe this would be worth a note in the figure caption.

 In the second paragraph of “Genome sequencing report” you stated uncertain regions to be in “chromosome 8, 9 and 11”. Please rephrase to “scaffold 8, 9 and 11”.

 In section “Genome assembly” you state “Pretext” but this should be either “rapid curation pipeline” (more precise) or “PretextView” (as in Table 3).

Are sufficient details of methods and materials provided to allow replication by others?

No

Is the rationale for creating the dataset(s) clearly described?

Yes

Are the datasets clearly presented in a useable and accessible format?

Yes

Are the protocols appropriate and is the work technically sound?

Yes

Reviewer Expertise:

Bioinformatics, de novo genome assembly and annotation

I confirm that I have read this submission and believe that I have an appropriate level of expertise to confirm that it is of an acceptable scientific standard, however I have significant reservations, as outlined above.

# Reviewer report: Reviewer response for version 1

**DOI:** 10.21956/wellcomeopenres.21139.r59819

**Published:** 2023-06-13

**Authors:** Chen Zhiteng

**Affiliations:** 1 Jiangsu University of Science and Technology, Zhenjiang, Jiangsu, China

The work presents a genome assembly from Isoperla grammatica, which is an important resource for the genomics of Plecoptera. However, the following comments should be addressed before acceptance for indexing.

1. In first paragraph of Background, please cite a reference for the distribution.
2. In Background, there is too much information on the biology. Please add information on genomics and genetics of I. grammatica, such as how many genomes of the family Perlodidae have been sequenced.
3. In the results, there is no information on the mitochondrial genome. Please clarify it, and compare with the published mitogenomes of I. grammatica in GenBank.
4. Figure 5, the boundary of the chromosomes is not clear, please check.

Are sufficient details of methods and materials provided to allow replication by others?

Yes

Is the rationale for creating the dataset(s) clearly described?

Yes

Are the datasets clearly presented in a useable and accessible format?

Yes

Are the protocols appropriate and is the work technically sound?

Yes

Reviewer Expertise:

Taxonomy, genomics, phylogeny

I confirm that I have read this submission and believe that I have an appropriate level of expertise to confirm that it is of an acceptable scientific standard, however I have significant reservations, as outlined above.
